# High-Sensitivity and Long-Life Microchannel Plate Processed by Atomic Layer Deposition

**DOI:** 10.1186/s11671-019-2983-1

**Published:** 2019-05-06

**Authors:** Weiwei Cao, Bingli Zhu, Xiaohong Bai, Peng Xu, Bo Wang, Junjun Qin, Yongsheng Gou, Fanpu Lei, Baiyu Liu, Junjiang Guo, Jingping Zhu, Yonglin Bai

**Affiliations:** 1 Key Laboratory of Ultrafast Photoelectric Diagnostic Technology, Xi’an Institute of Optics and Precision Mechanics, Chinese Academy of Sciences, Xi’an, 710119 China; 2 Key Laboratory for Physical Electronics and Devices of the Ministry of Education and Shaanxi Key Laboratory of Information Photonic Technique, Xi’an Jiaotong University, Xi’an, 710049 China; 3 University of Chinese Academy of Sciences, Beijing, 100091 China; 4 State Key Laboratory of Transient Optics and Photonics, Xi’an Institute of Optics and Precision Mechanics, Chinese Academy of Sciences, Xi’an, 710119 China; 5 Collaborative Innovation Center of Extreme Optics, Shanxi University, Taiyuan, 030006 Shanxi China

**Keywords:** Microchannel plate (MCP), Atomic layer deposition (ALD), Thin film, High stability, Long lifetime

## Abstract

As a key component of electron multiplier device, a microchannel plate (MCP) can be applied in many scientific fields. Pure aluminum oxide (Al_2_O_3_) as secondary electron emission (SEE) layer were deposited in the pores of MCP via atomic layer deposition (ALD) to overcome problems such as high dark current and low lifetime which often occur on traditional MCP. In this paper, we systematically investigate the morphology, element distribution, and structure of samples by scanning electron microscopy (SEM) and energy disperse spectroscopy (EDS), respectively. Output current of different thickness of Al_2_O_3_ was studied and an optimal thickness was found. Experimental tests show that the average gain of ALD-MCP was nearly five times better than that of traditional MCP, and the ALD-MCP showed better sensitivity and longer lifetime.

## Introduction

Microchannel plate (MCP) is a compact electron multiplier of high gain [1–3]. It is a key component in MCP-PMT, night vision devices, electron microscopy, X-ray framing camera, and so on [4–7]. It has been used in a wider range of particle and photon detection applications, such as ions, electron, neutrons, X-ray, and UV ray [8–11]. Microchannel plate is a thin two-dimensional array composed of several millions of ultra-thin conductive glass pores from 4 to 25 μm in diameter and 0.2 to 1.2 mm in length. MPC has three principle structure characteristics—bias angle, ratio of channel length to channel diameter, and open area ratio (ratio of the total open area to the entire effective area). In general, the bias angle ranges from 5 to 15°. The ratio of channel length to channel diameter is about 20:1 to 100:1, and the open area ratio about 60 to 80%. Traditional MCP is made of lead silicate glass and fabricated by drawing, stacking, fusing, slicing, etching, and hydrogen reduction. After hydrogen reduction chemical processing, conductive layer and secondary electron emission (SEE) layer are generated in the pore. When an electron or radiation enters a channel, secondary electrons are emitted from the SEE layer and those electrons are accelerated by an electric field developed by a voltage *V*_D_ applied on the conductive layer. Finally, further secondary electrons were produced in this way and electron amplification was realized. Although traditional MCP has been widely used in many fields, there are several drawbacks [12]. Firstly, high-noise chemical etching increases Ra on the inner surface of the pore, noise factor increases, and S/N reduces when the photoelectrons are multiplied. Secondly, vacuum baking and electron scrubbing result in MCP surface element variation and reduce the extracted charge and gain of MCP. Thirdly, electrical resistance and the secondary electron emission properties cannot be adjusted independently. Because the causes of drawbacks are different and the production process of traditional MCP is complex, it is difficult to overcome all disadvantages at the same time by adjusting process parameters.

As atomic layer deposition (ALD) technology is applied in more and more research area [13–17], some researchers proposed deposited conductive layer and SEE layer inside the channels to optimize the performance of traditional MCP [18–23]. ALD is a thin-film deposition technique in which a film is grown on a substrate by exposing its surface to alternate gaseous species. ALD is considered as one deposition method with great potential for producing very thin, conformal films with control of the thickness and composition of the films possible at the atomic level [24, 25].

There are many advantages applying the ALD technology in MCP post-processing. Silicon dioxide (SiO_2_) is the main secondary electron emission material in traditional MCP [26] and has a low secondary electron yield (SEY). If we deposited a high SEE material on the channel wall, the gain of MCP could be improved. Because of etching process, inner channel surfaces of traditional MCP stay a higher surface roughness and have lots of nanoscale holes. The nanoscale holes adsorb gases and other pollutants which are hard to clean. When accelerated, electrons hit absorbents, which are ionized and accelerated by the electric field. The accelerated ions ionized more and more absorbents and consume extra electron of the MCP. Because of the lifetime of MCP decided by total quantity of electric charge, the existence of absorbents leads to a shorter lifetime. In the meanwhile, the electric wash process is necessary to ensure a high vacuum. More absorbents need longer electric wash time and cost more electric charge, further reducing MCP lifetime. If we deposited several nanometers high SEE material on the inner channel surface, parts of nanoscale holes could be filled up to improve surface roughness, then to have a longer lifetime.

In this paper, Al_2_O_3_ as SEE material was prepared by ALD technique in the pores of MCP with 15-mm length of side. The morphology, element distribution, structure of ALD deposited oxide thin film, and performance of ALD-MCP were systematically investigated.

## Experimental and Calculation Methods

**Fig. 1:**
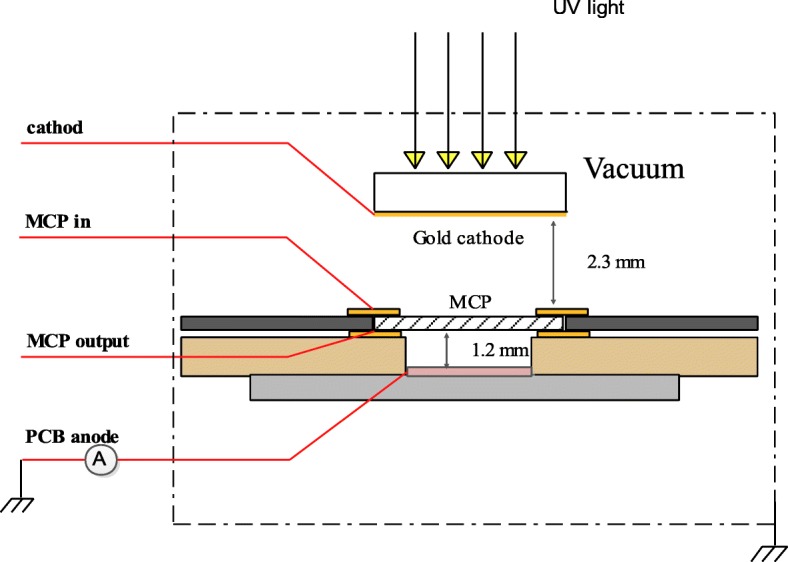
The schematic of the experiment setup

The schematic of the experiment setup is shown in Fig. 1. The system consists of gold cathode, MCP, and PCB anode and is placed in a vacuum chamber evacuated to 2 × 10^−4^ Pa. Voltages for all electrodes are supplied by a multichannel high voltage power supply and are fed into the vacuum chamber by high voltage feedthroughs. There is a picoammeter between PCB anode and the ground to measure the MCP output current. An attenuated mercury lamp acts as ultraviolet light source when we measure the MCP output current. The mercury lamp without attenuation serves as ultraviolet light source when we accelerate the life testing of microchannel plate.

**Fig. 2:**
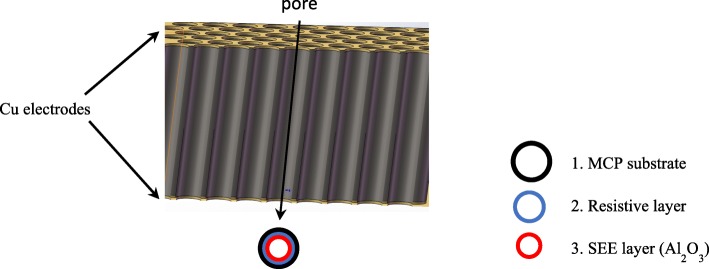
Structure schematic diagram of ALD-MCP

A commercial hot-wall atomic layer deposition system was used to prepare nano-oxide thin films onto MCP inner channel surface (as shown in Fig. 2) and silicon. The samples deposited on silicon are used to measure secondary emission yield. The polished silicon substrates were ultrasonically cleaned in an acetone/ethanol/hydrofluoric acid/DI water and then placed in an ALD chamber waiting for deposition. The bare MCPs (thickness = 1.2 mm, pore size = 24 μm, aspect ratio = 40, bias angle = 10°) were heated to 200 °C for 1 h to growing nano-oxide thin films. According to the paper [27, 28], it is harder to control the thickness and composition of materials on MCP than on planar substrate. Two approaches were adopted to make thickness and composition as far as possible to be uniformly distributed in the pores of MCP. One is extending precursors for each ALD cycle (sample F). The other is using a stop flow model (sample G), which precursors pulse enter and diffuse in the hot-wall chamber for several seconds, then pumped out and purged by carrier gas.

For second secondary electron emission layer deposition, Al_2_O_3_ was performed using TMA and deionized water as Al and oxidant precursor, respectively. Ultrahigh purity nitrogen was used as a carrier and purge gas. The Al_2_O_3_ ALD was performed using separate TMA and H_2_O exposure with sequence TMA/N_2_/H_2_O/N_2_ (0.05/10/0.05/10s). The square MCP with 15-mm length of side coated approximately 4 nm (sample B), 6 nm (sample C), 8 nm (samples D and H), 10 nm (sample E), and 60 nm Al_2_O_3_ (sample F). The sample G was performed using separate TMA and H_2_O exposure with sequence TMA/Stop/N_2_/H_2_O/Stop/N_2_ (0.05/3/20/0.05/3/20s) for 600 periods. One circular MCP (50 mm in diameter) was divided into two parts; one part was bared and the other part was covered by two pieces of semicircular silicon wafer, to obtain a MCP which is half with ALD process and half unprocessed. The details of experimental parameters are listed in Table 1. The surfaces of MCP samples were examined by scanning electron microscopy (SEM). The film elemental composition was measured by cross-sectional SEM method (EDS). After ALD functionalization, the copper layer with 200 nm as electrodes was prepared on both MCP sides by evaporation system for MCP electrical characterization and life testing.

**Table 1:** Details of experimental samples for ALD

	Condition	Description
Sample A	Uncoated	Samples A, B, C, D, and E to confirm the best thickness of deposition layer
Sample B	Coated 4-nm ALD Al_2_O_3_	
Sample C	Coated 6-nm ALD Al_2_O_3_	
Sample D	Coated 8-nm ALD Al_2_O_3_	
Sample E	Coated 10-nm ALD Al_2_O_3_	
Sample F	Coated 60-nm ALD Al_2_O_3_ Extending precursors model	Samples F and G to confirm which deposition model is better
Sample G	Coated 60-nm ALD Al_2_O_3_ Stop flow model	
Sample H	Half coated 8-nm ALD Al_2_O_3_ Half no coated	

## Results and Discussion

As shown in Fig. 1, the principle of MCP gain test is shown below. Ultraviolet photon arrives at gold cathode and was converted to photoelectron by photoelectric effect. Photoelectron is accelerated by voltage between cathode and MCPin and obtains a primary electron energy. Then, photoelectron with primary electron energy amplified by MCP and output electron clouds to PCB anode. At last, electrons flow to the ground and the output current was measured by picoammeter. The output current of MCP is ordered by bias voltages between different electrodes. In order to determine voltages for every electrode, sample A was assembled and voltage between MCPin and MCPout was set to a fixed value; then, change voltages of cathode and anode to get an optimum value.

**Fig. 3:**
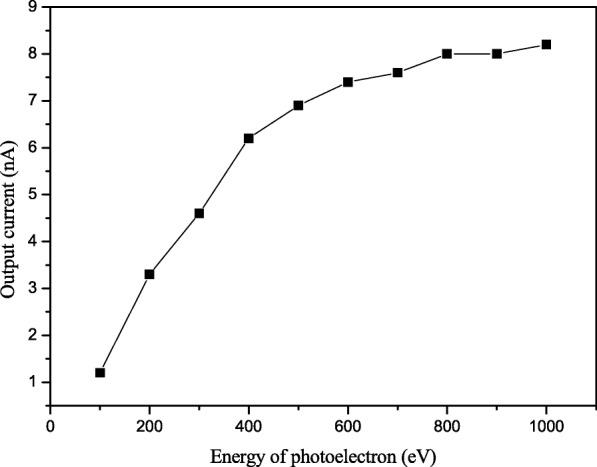
Bias voltage between cathode and MCPin as function of MCP output current

The output current of MCP as function of energy of photoelectron (the primary electron energy before enter MCPin) is shown in Fig. 3 when the bias voltage setting for MCP is 1400 V. The output current of MCP approximate increases linearly as energy of photoelectron less than 400 V and enters a plateau at energy larger than 400 eV. This can be attributed to the fact that the SEY of silicon dioxide is particularly low at lower incident electron energy and once the SEY is near the maximum, the output current becomes stable.

**Fig. 4:**
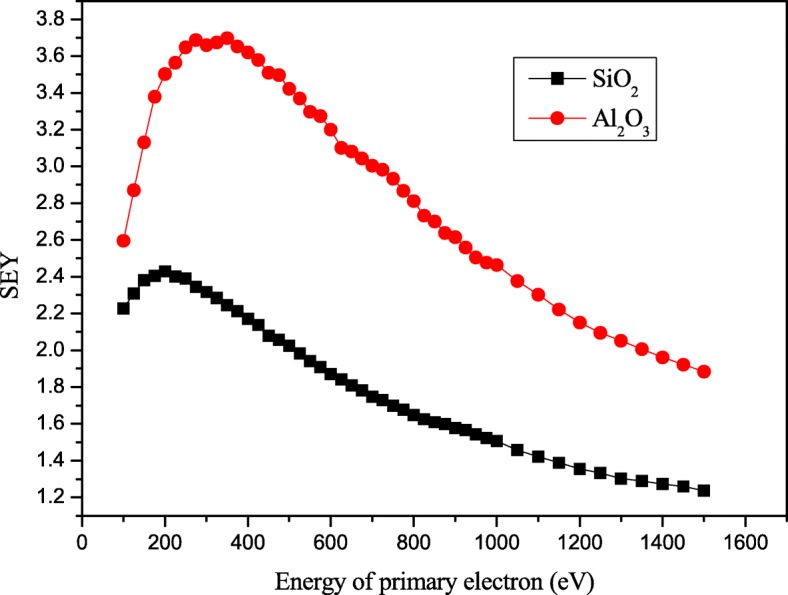
Secondary electron yield (SEY) of SiO2 ALD Al2O3

Figure 4 shows the SEY curve of Al_2_O_3_ and SiO_2_. In the figure, the SEY of Al_2_O_3_ is increasing with the voltage and gets the largest value of 3.6 at 400 V, and this tendency approximately corresponds with Fig. 3. In the Al_2_O_3_ SEY curve, the SEY value gets down larger than 400 eV. But as shown in Fig. 3, the output current is still increasing when the bias voltage is above 400 V. This can be explained by the open area ratio of MCP. The MCP we used has an open area ratio about 60%; it means when photoelectrons arrive at the MCPin, 40% of them cannot enter into channels and would be reflected by the top surface of MCP. When the voltage between cathode and MCPin increases, electric field would re-accelerate the 40% electrons and enter the channel again.

**Fig. 5:**
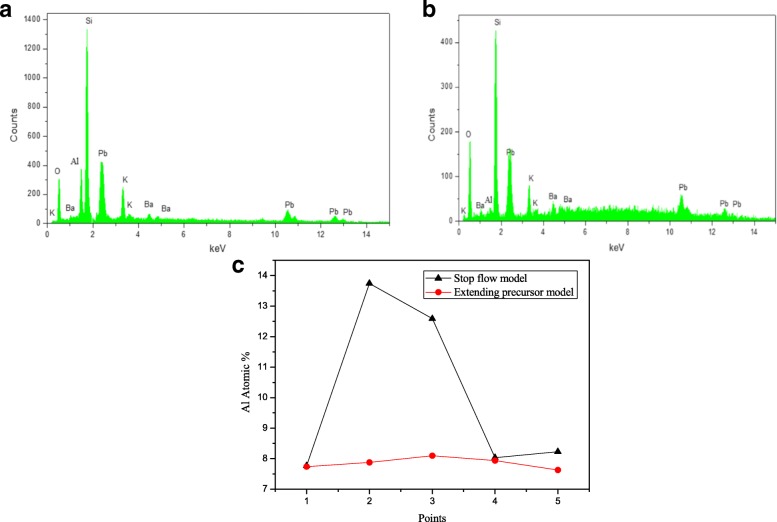
Spectra and elemental composition of cross-sectional MCP samples. **a** Spectra and elemental composition of ALD MCP. **b** Spectra and elemental composition of uncoated MCP. **c** Al distribution of samples deposited by stop flow model and extending precursor model.

Because of the uniformity of SEE layer can affect the uniformity of MCP imaging detector, so the uniformity of SEE layer is a key factor to characterization ALD-MCP. Figure 5 shows the spectra and elemental composition of cross-sectional MCP samples processed by extending precursor model and stop flow model. The Al distribution is characterized by EDS at five locations along the pore inner surface. In order to reduce the EDS measure error, the element distribution data was measured by two thicker samples, sample F and sample G, which deposited 60 nm Al_2_O_3_. In Fig. 5a, b, the elementary composition of coated and uncoated MCPs was measured to exclude the influence of substrate on the distribution of Al. The amount of Al is lower than 1% in the substrate and too small to affect the final experiment results. Al distribution of samples deposited by stop flow model and extending precursor model is shown in Fig. 5c. Al content at different locations signified that the elements are more uniformly distributed in the pore inner surface which sample deposited by extending precursors model. This also implies that the ALD technique is capable of depositing homogeneous nano-oxide thin film on substrates with complex structure. The element distribution of sample deposited by stop flow model shows a bad uniform. The top and bottom surfaces of MCP have a low atomic percent, while the middle of the channel has a high atomic percent. This is probably because the precursors on the surface were easy to purge and get an atomic deposition layer. In the middle of channel, precursors were hard to purge and get a vapor deposition instead of atomic deposition.

**Fig. 6:**
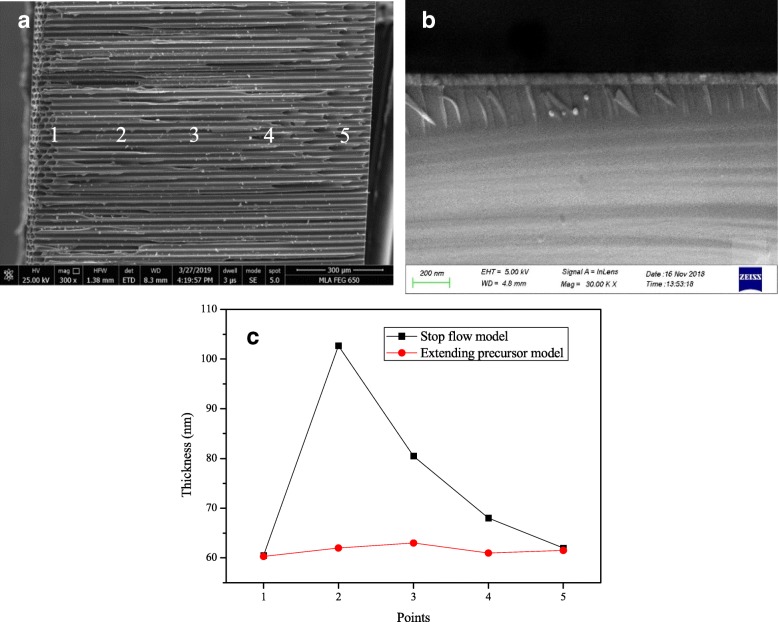
Cross-sectional SEM pictures of MCP. **a** Cross-sectional SEM pictures of ALD-MCP samples. **b** Cross-sectional SEM pictures of Al2O3 layer on the surface of inner channel. **c** Thickness of SEE layer at different location measured with SEM

The thickness with SEM might be a better option to confirm the uniformity. So the thickness of SEE layers deposited on the inner surface of MCP pores was measured by SEM and is summarized in Fig. 6. Thickness of five different points along one pore, as shown in Fig. 6a, was directly measured by SEM. The thickness curves of different deposition model are shown in Fig. 6c, which basically matches with the Al distribution as in Fig. 5c.

**Fig. 7:**
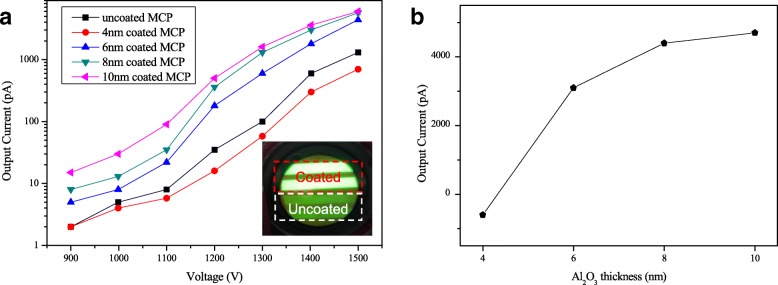
Output current and image of traditional MCP and coated ALD-MCP. **a** Output current of different coated thickness of Al2O3 on MCP and photograph of a phosphor screen illuminated by half coated 8-nm Al2O3 and half uncoated. **b** Output current of coated subtract uncoated MCP as function of Al2O3 thickness

After copper electrodes were prepared on both sides of MCP, the electrical characterization measured by a system is shown in Fig. 1. Figure 7 shows output current and image of traditional MCP and coated ALD-MCP. Figure 7a shows the output current as a function of different thickness of Al_2_O_3_. In Fig. 7b, as the film thickness increases from 6 to 10 nm, the output current of coated subtract uncoated MCP significantly grows first and then keeps stable. The ALD-MCPs with 8-nm and 10-nm Al_2_O_3_ coating got more than five times output current than conventional MCP. It means that 8-nm thick Al_2_O_3_ is optimal for MCP application. This is caused by the ALD-MCP which has a higher SEY material which we can see from Fig. 3. As is shown in (a), when coated 4 nm on inner channel surface of MCP, the output current is lower than the uncoated MCP. This probably because there is no continuous material and lots of defects exist when the thickness of Al_2_O_3_ is below 4 nm. Electrons recombination occurs in defects to reduce the number of secondary electrons and lead to a lower output current than uncoated MCP.

After we deposited 8-nm Al_2_O_3_ on half part of sample H, there are four copper strips deposited on sample H and a phosphor screen instead of PCB anode to collect the output electrons. As shown in Fig. 7b, half coated MCP has a brighter image than uncoated MCP. This is consistent with electrical characterization.

Sample A and sample D were continuously illuminated with an ultraviolet mercury lamp for a lifetime testing. In order to accelerate the life testing, high power ultraviolet mercury lamp without attenuation was used. MCPs were operated with large output currents and stay at a saturation level for several days. As shown in Table 2, the dark current and output current at a low illumination level were measured before and after lifetime testing. Before testing, dark current of traditional MCP was 1.0 pA and the ALD-MCP was 1.2 pA. The higher dark current of ALD-MCP was just because the substrates of MCPs were traditional MCPs and the ALD-MCP was coated by a high SEY material. After lifetime testing, the dark current of ALD-MCP performs better while they showed similar performance before lifetime testing. According to Table 2, output current of traditional MCP dropped about 50% after high power illumination, while ALD-MCP shows a better behavior and output current kept around 6nA.

**Table 2:** Dark current and output current measured before and after lifetime testing

	Dark current before lifetime testing (pA)	Dark current after lifetime testing (pA)	Output current before lifetime testing (pA)	Output current after lifetime testing (pA)
Traditional MCP	1.0	6.5	1300	630
ALD-MCP	1.2	1.5	5700	6000

## Conclusions

The morphology, composition, and structure of nano-oxide thin films Al_2_O_3_ prepared via atomic layer deposition were investigated. The thickness uniformities in the channel deposited by extending precursor model and stop flow model were measured. Al contents and film thickness at different locations along the channels signified that extending precursor can obtain a better uniformity for a MCP with pore size of 24 μm and aspect ratio of 40. We have evaluated a MCP testing system and observed that the bias voltages between cathode and MCP top surface can affect output current. The electrical properties and lifetime measurements were studied. The electrical measurements results showed when film thickness increases from 6 to 10 nm, the output current increased and speed decreases. And ALD-MCPs which are coated more than 8-nm Al_2_O_3_ got about five times output current than traditional MCPs and have a better performance of lifetime.

## Abbreviations

ALD: Atomic layer deposition
ALD-MCP: Microchannel plate processed by atomic layer deposition
EDS: Energy disperse spectroscopy
MCP: Microchannel plate
SEE layer: Secondary electron emission (SEE) layer
SEM: Scanning electron microscopy
SEY: Secondary electron yield
